# Drug Repurposing of the Alcohol Abuse Medication Disulfiram as an Anti-Parasitic Agent

**DOI:** 10.3389/fcimb.2021.633194

**Published:** 2021-03-11

**Authors:** Debbie-Ann Shirley, Ishrya Sharma, Cirle A. Warren, Shannon Moonah

**Affiliations:** ^1^ Division of Pediatric Infectious Diseases, Department of Pediatrics, University of Virginia, Charlottesville, VA, United States; ^2^ Division of Infectious Diseases & International Health, Department of Medicine, University of Virginia, Charlottesville, VA, United States

**Keywords:** drug development, parasites, protein degradation, drug repurposing and repositioning, disulfiram (Antabuse)

## Abstract

Parasitic infections contribute significantly to worldwide morbidity and mortality. Antibiotic treatment is essential for managing patients infected with these parasites since control is otherwise challenging and there are no vaccines available for prevention. However, new antimicrobial therapies are urgently needed as significant problems exist with current treatments such as drug resistance, limited options, poor efficacy, as well as toxicity. This situation is made worse by the challenges of drug discovery and development which is costly especially for non-profitable infectious diseases, time-consuming, and risky with a high failure rate. Drug repurposing which involves finding new use for existing drugs may help to more rapidly identify therapeutic candidates while drastically cutting costs of drug research and development. In this perspective article, we discuss the importance of drug repurposing, review disulfiram pharmacology, and highlight emerging data that supports repurposing disulfiram as an anti-parasitic, exemplified by the major diarrhea-causing parasite *Entamoeba histolytica*.

## Introduction

Protozoan parasites cause more than one million deaths annually, disproportionately affecting poverty-stricken areas of the world (Murray et al., 2012; Torgerson et al., 2015; De Rycker et al., 2018). Of these, *Plasmodium falciparum*, which causes human malaria, is responsible for over 618,000 of these deaths each year, while *Leishmania*, which causes visceral leishmaniasis, a febrile disease marked clinically by fatal visceromegaly if untreated, falls in line after with the second highest mortality among tropical diseases (Weiss et al., 2019). *Entamoeba histolytica*, which causes amebic colitis and amebic liver abscesses, infects millions worldwide and kills more than 50,000 people each year (Lozano et al., 2012; Shirley et al., 2018). Other protozoan parasites, such as *Toxoplasma gondii*, which infect up to a third of the world’s population and causes devastating congenital infection, continue to pose a serious threat to health, especially among the world’s poorest populations. The global, social and economic impact of these neglected diseases including trypanosome infections is untold. Antibiotic treatment is essential for managing patients infected with these protozoan parasites, but therapeutic options are severely limited due to the cost, poor efficacy, drug resistance and toxicity associated with currently available therapies. Discovery of new treatment is therefore urgently needed (De Rycker et al., 2018).

## Significance of Amebiasis and Why New Treatment Options Are Needed

To examine in more detail some of the limitations faced in treating protozoan parasitic disease, the case of amebiasis provides a good example. Amebic colitis is a leading cause of severe diarrhea worldwide and is listed among the top 15 causes of diarrhea in the first 2 years of life and a top seven cause of dysentery in children under five years (Kotloff et al., 2013; Liu et al., 2016; Shirley et al., 2018). Multiple areas throughout the world continue to observe prevalence rates of amebiasis of over 10% (Shirley et al., 2018). Severe forms of amebic colitis are associated with high mortality, and on average around half with severe colitis die (Shirley and Moonah, 2016). Immigration, travel and sexual transmission are leading to re-emergence of amebiasis in developed settings (Hung et al., 2012; Cordel et al., 2013; Van Den Broucke et al., 2018).

Only one class of drugs (the nitroimidazoles, such as metronidazole) are available to effectively treat invasive forms of disease, with little progress made in new drug development over the past 60 years (Marie and Petri, 2013; Gonzales et al., 2019). No alternative therapies exist for those who experience toxicity from metronidazole, such as neurologic and dermatologic effects (Woodruff et al., 2002; Kumar et al., 2013; Mazumdar and Shome, 2014; Sørensen et al., 2018; Cappellari et al., 2020). This is illustrated in a recent report from Japan of two patients with ulcerative amebic colitis who received sub-standard therapy due to not being able to take metronidazole because of side effects (Yamamoto et al., 2020). Additionally, there are no treatment options if metronidazole resistance develops (Shirley et al., 2018; Kumanan et al., 2020). Clinical use of metronidazole, both appropriate and inappropriate, is widespread with metronidazole ranking amongst the most commonly used antibiotics for diarrhea worldwide. (Rogawski et al., 2017). There are concerns that high antibiotic pressure will give rise to resistance with amebiasis. Resistance to metronidazole has already been documented among other similar anaerobic protists *Trichomonas vaginalis* and *Giardia lamblia* (Schwebke and Barrientes, 2006; Kirkcaldy et al., 2012; Paulish-Miller et al., 2014). Along with the ease with which resistant amebic strains can be generated in the laboratory raises concerns that it is only a matter of time before drug resistant amebiasis emerges (Leitsch, 2015; Ehrenkaufer et al., 2018). We are ill-prepared for scenarios like resistance; therefore, new anti-amebic therapies are called for (Shirley et al., 2019). Innovative solutions, like drug repurposing will help meet this need.

## The Importance of Drug Repurposing

Drug repurposing, also known as drug repositioning, reprofiling, or re-tasking, involves identifying new uses of already approved, discontinued, or investigational drugs outside of the original indication. New drug discovery and development is a slow, arduous, and costly process that is further hampered by high failure rates. As protozoan parasitic infections are diseases of the poor, global investments are inadequate, though improving, for disease control. Repurposing of old drugs hence serves as an appealing alternative strategy to lower cost and save time. It is estimated that the cost of repurposing a drug is about one-tenth that of the 2-3 billion dollar investment commonly required in the development of a new drug. The regulatory process is additionally simplified if the safety, formulation, and pharmacokinetic data essential in preclinical and early clinical safety studies to bring a drug to market have been completed, allowing resources to be focused on the more advanced phases of efficacy trial studies. Often bulk manufacturing has already been established as well (Andrews et al., 2014; Farha and Brown, 2019; Pushpakom et al., 2019). Well-known examples of repurposed drugs include sildenafil, an antihypertensive drug repurposed for erectile dysfunction treatment and miltefosine, an anti-neoplastic agent repurposed for leishmaniasis (Fink et al., 2002; Braga, 2019). Here we share the perspective of repurposing disulfiram as an anti-parasitic agent. Other compounds with potential anti-amebic effects have been well-reviewed elsewhere (Debnath et al., 2013; Andrade and Reed, 2015; Jeelani and Nozaki, 2016; Martínez-Castillo et al., 2018; Nagaraja and Ankri, 2019).

## Disulfiram

Disulfiram, or tetraethylthiuram disulfide (Antabuse), is an FDA-approved drug that has been used to treat alcohol dependence for the past seven decades, with well-established pharmacokinetic properties, safety, and tolerance (**Table 1**) (Johansson, 1992; Suh et al., 2006).

**Table 1:** Overview of Disulfiram.

Name	Disulfiram, or tetraethylthiuram disulfide (Antabuse)
**FDA approval**	1951
**FDA approved indication**	Alcohol Use Disorder
**Mechanism of Action**	Aldehyde dehydrogenase inhibitor
**Potential repurposed indications** **(not FDA-approved)**	Anti-neoplastic Anti-microbial
**Metabolites**	Diethyldithiocarbamate, diethyldithiomethylcarbamate, carbon disulfide, dimethylamine, and divalent metal complexes such as zinc ditiocarb (ZnDTC)
**FDA prescribing**	250 (125 – 500) mg/day
**Route**	By mouth
**Elimination**	Metabolites excreted mainly by kidney, feces and lung
**Adverse events**	Disulfiram-alcohol reaction, hepatotoxicity, neuropathy, psychosis
**Examples of drug interactions**	Metronidazole, alcohol and alcohol-containing medications, phenytoin, anticoagulants e.g. warfarin, amitriptyline, isoniazid, diazepam
**Contra-indications**	Concurrent alcohol use, hypersensitivity to disulfiram or thiuram derivatives
**Special populations**	Safety in pregnant or nursing mothers and children has not been established.

FDA, US Food and Drug Administration.

The discovery of disulfiram relates back to a series of chance discoveries. Initially used as a compounding agent to accelerate the manufacturing process of rubber production in the 18^th^ century, it was noted in 1937 by an American chemical plant physician that workers exposed to disulfiram became intolerable to alcohol consumption (Suh et al., 2006; Kragh, 2008). Later, Danish researchers exposed to disulfiram while investigating its vermicidal properties, noted that disulfiram changed the effect of alcohol to an unpleasant experience, prompting collaborative efforts to investigate disulfiram as a therapeutic agent aimed to deter alcohol consumption. Disulfiram was subsequently approved by the US Food and Drug Administration for treatment of alcoholism in 1951 (Suh et al., 2006; Kragh, 2008; De Sousa, 2019). It is now understood that the disulfiram-alcohol reaction is due to the inhibition of aldehyde dehydrogenase, increasing the levels of acetaldehyde in the blood and leading to disagreeable physical effects such as flushing, sweating and headache upon consuming alcohol. Collectively, studies evaluating the efficacy of disulfiram have yielded variable results, likely related to limitations in study methods, such as small sample sizes, lack of randomization, unmasked design, short follow-up periods, and inability to measure treatment adherence. Disulfiram remains a recommended deterrent that can be safely used in motivated and well-informed patients wishing to abstain from alcohol (Skinner et al., 2014; Reus et al., 2018).

### Dosage and Frequency

Using the FDA-approved indication of management of chronic alcohol use, patients may be given a dose of 250 mg, to be taken by mouth once daily, with a maximum dosage of 500 mg/day. Newer off label uses describe administration of disulfiram 500 mg daily plus zinc gluconate 50 mg three times daily or copper gluconate 2 mg three times daily in combination therapy (Ekinci et al., 2019).

### Absorption, Distribution, and Excretion

Approximately 80 – 95% of ingested disulfiram is absorbed and is more efficiently dissolved in the gastric juice as an effervescent tablet; fractions that are unabsorbed are excreted. In the body, disulfiram is rapidly metabolized to diethyldithiocarbamic acid (ditiocarb, DTC) which then quickly forms diethylthiocarbamic acid methyl ester (MeDTC) or is broken down into carbon disulfide and dimethylamine. MeDTC is the metabolite mainly responsible for the alcohol effects by inhibiting acetaldehyde dehydrogenase. DTC is a strong metal chelating substance that forms complexes with metal ions, for example in the presence of the metal ion zinc, forms zinc-ditiocarb complex (ZnDTC). Disulfiram is also reduced to DTC in the stomach where it can form metal ion complexes in the gastrointestinal tract. DTC-metal complexes have a relatively long half-life, are widely distributed throughout the body, including penetration of the blood-brain barrier, and are found in urine, bile, and feces (Johansson, 1992; De Sousa, 2019).

### Safety

Disulfiram is a safe and well-tolerated medication. When side effects are reported, they are generally mild. The higher doses initially used for clinical treatment were associated with more side effects. In the absence of alcohol, disulfiram administered at the currently recommended dosages is well tolerated for months and even years. In case of intolerance, the dose of disulfiram can be lowered (Børup et al., 1992; Brar et al., 2004; Skinner et al., 2014). When taken with alcohol, the disulfiram-alcohol reaction produces serious adverse effects. Therefore, alcohol consumption must be avoided while taking disulfiram. Cutaneous alcohol absorption from hand sanitizers and topical products such as perfumes should not result in a disulfiram-alcohol reaction and risk through inhalation can be mitigated by dispersal of fumes (Brewer and Streel, 2020).

## Repurposing Disulfiram as an Anti-Amebic Agent

As the years go by, new medical uses for disulfiram are being discovered beyond the inhibition of aldehyde dehydrogenase. As described above, disulfiram is metabolized to ditiocarb which then forms metal complexes. Ditiocarb-metal complexes have anti-cancer effects and are safely given *in vivo* as oral disulfiram plus copper or zinc supplements. Multiple completed, active, or recruiting clinical trials evaluating disulfiram in combination with divalent metal ion supplement zinc or copper for cancer therapy have shown excellent safety profile (Brar et al., 2004; Grossmann et al., 2013; Nechushtan et al., 2015; Skrott et al., 2017; Ekinci et al., 2019).

**Figure 1:**
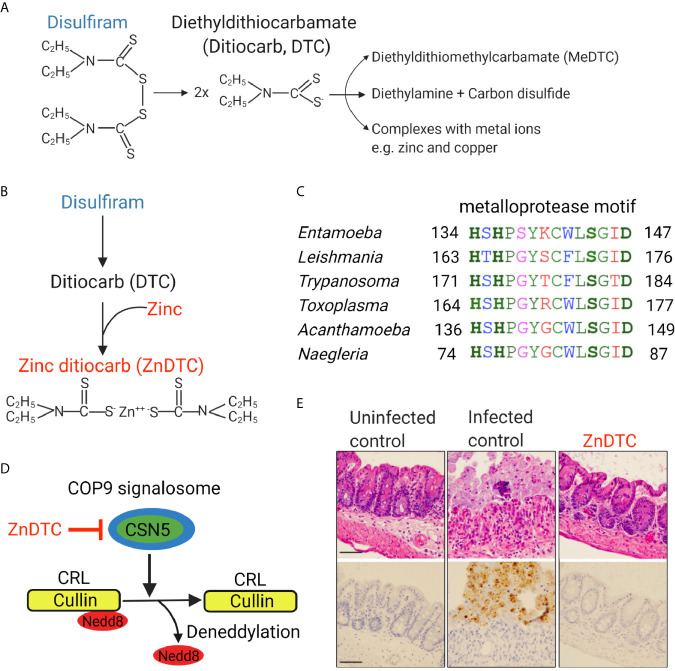
Zinc ditiocarb (ZnDTC), a main metabolite of disulfiram, is potently active against *E. histolytica* parasites in a preclinical animal model. **(A)** Disulfiram and its metabolites. **(B)** ZnDTC is formed from the combination of disulfiram and zinc supplement. **(C)** Multiple sequence alignment of the conserved metalloprotease motif of COP9 signalosome subunit 5 (CSN5) from *E*. *histolytica* and other pathogenic parasites (EHI_050500, TGARI_308590, TcCLB.507083.60, LBRM2903_160015100, ACA1_074760, NF0060740). Identical (green), conserved (blue), semi-conserved (pink) and non-conserved residues (red). **(D)** Schematic of how ZnDTC inhibits cullin deneddylation by the COP9 signalosome. **(E)** Representative H&E staining and immunohistochemical analysis of the cecum of infected mice after 5 days of treatment. Specific anti–*E. histolytica* antibody was used for immunohistochemical staining of trophozoites (brown). Numerous parasites in the infected control, absent in the ZnDTC treated mice and uninfected control. The treatment group received 50 mg/kg disulfiram plus 1 mg/kg zinc gluconate. Panels are reproduced from (Ghosh et al., 2020) with permission.

A new preclinical study using a mouse model that simulates human amebic colitis showed that oral disulfiram combined with zinc was highly effective in clearing parasites (**Figure 1**) (Ghosh et al., 2020). Zinc-ditiocarb complex (ZnDTC) had high potency and was active against *E. histolytica* parasites at low nanomolar concentrations (Ghosh et al., 2020), significantly below the serum and tissue level achieved with disulfiram therapy at recommended doses (Johansson, 1992; Skrott et al., 2017), and 1000-fold-lower less than the EC_50_ of metronidazole, the current drug of choice to treat amebiasis.

The Ubiquitin-Proteasomal System (UPS) is responsible for the vast majority of protein degradation within the cell and is conserved in all eukaryotes including protozoans. Disruption of the proteasomal pathway results in accumulation of unwanted and toxic proteins, leading to cell death. Because proteasome-mediated degradation is essential for parasite survival, this pathway has become an attractive drug target for protozoan parasites (Khare et al., 2016; Li et al., 2016). The anti-amebic activity of ZnDTC was shown to involve inhibition of the ubiquitin-proteasome pathway (Ghosh et al., 2020).

E3 ubiquitin ligases catalyze the ubiquitination of proteins destined for proteasomal degradation (Cromm and Crews, 2017; Wertz and Wang, 2019). Cullin proteins form the base on which cullin-RING E3 ubiquitin ligases (CRLs) assemble and CRLs make up the majority of E3 ligases. Regulation of CRL activity involves the removal of the ubiquitin-like protein called Nedd8 from cullin proteins by a process called deneddylation. The COP9 signalosome is a multi-subunit protein complex that is responsible for the enzymatic removal of Nedd8 protein from cullins (Cavadini et al., 2016; Mevissen and Komander, 2017). COP9 subunit 5 (CSN5) contains a metalloprotease motif and forms the catalytic core of the COP9 enzyme complex. CSN5 was found to be encoded by *Entamoeba histolytica* as well as many other clinically relevant protozoans including *Trypanosoma, Leishmania, Toxoplasma*, and *Naegleria* (Ghosh et al., 2018; Ghosh et al., 2020). COP9 activity is essential for *Entamoeba histolytica* survival. Through computational, genetic, and protein degradation studies, ZnDTC was found to block the metalloprotease activity of CSN5 which disrupted COP9 signalosome deneddylating activity. This caused cullin to be trapped in a neddylated state, disrupting proteasome-mediated degradation, leading to protein accumulation and ultimately cell death (Ghosh et al., 2020).

Additionally, disulfiram may inhibit the glycolytic pathways of *E. histolytica* (Pineda et al., 2013; Pineda et al., 2015). It should be noted that while disulfiram by itself has shown direct activity *in vitro*, the drug is rapidly metabolized and therefore would be less effective *in vivo* if given alone. The metabolites such as ZnDTC metal complexes are stable and would be more practical for clinical use. The safety profile along with preclinical efficacy supports further studies to explore the repurposing of disulfiram for amebiasis treatment, which could be the first new amebic treatment available in over 60 years.

## Repurposing Disulfiram as an Anti-Parasitic Agent

While disulfiram, when used by itself, shows varying activity against parasites, *in vitro* studies have found a greater anti-parasitic potency of metabolites when complexed to divalent metal ions such as zinc (ZnDTC) (Peniche et al., 2015; Oliveira et al., 2019). It is plausible then that the metabolites of disulfiram, which remain for a longer duration than the rapidly metabolized disulfiram, may explain the broad range of targets described with this drug in a diverse range of pathogens (Sheppard et al., 2018; Frazier et al., 2019; Lee et al., 2019; Trautmann et al., 2020).

More specifically as an anti-parasitic, *in vitro* efficacy of the metabolite ZnDTC has also been demonstrated against other protozoan parasites *Trypanosoma* and *Leishmania* (Peniche et al., 2015; Oliveira et al., 2019; Ghosh et al., 2020). While the effect of zinc ditiocarb on other parasites is not yet known, COP9 producing protozoans should experience the same effect with ZnDTC (Ghosh et al., 2018; Ghosh et al., 2020). That said, further studies are needed to confirm this theory. Other anti-parasitic mechanisms of disulfiram that have been proposed include the inhibition of carbamate kinase and triosephosphate isomerase in *Giardia lamblia* (Galkin et al., 2014; Castillo-Villanueva et al., 2017). Inhibitory effects on growth of *Cryptosporidium parvum* and *Plasmodium falciparum* have also been shown (Scheibel et al., 1979; Sarwono et al., 2019). Therefore, ZnDTC might be able to treat a wide range of parasitic infections, one drug for many bugs. The established safety plus *in vivo* efficacy data support further investigation of ZnDTC, administered as repurposed disulfiram with zinc (Ekinci et al., 2019; Ghosh et al., 2020).

## Limitations Associated With Disulfiram Use

Alcohol consumption will negatively impact disulfiram adherence. Though, patients prescribed disulfiram need only to abstain from alcohol for 12 hours before initiation of use. Metronidazole too is associated with a similar “disulfiram-like reaction” and alcohol intake is contraindicated with metronidazole use (Cina et al., 1996; Alonzo et al., 2019), yet metronidazole remains one of the most widely used antibiotics worldwide. In addition, the treatment course for amebiasis is relatively short, therefore, only a predicted 5 to 10-day disulfiram course would be required. All these reasons provide support for disulfiram adherence.

In addition to adults, parasitic infections like amebiasis pose a global health threat to children. Disulfiram was developed and mainly used in adolescent and adult populations (Niederhofer and Staffen, 2003). Therefore, data on pediatric PK/PD properties are additionally needed for age-appropriate dosage.

## Conclusion

Protozoan parasites, such as *E. histolytica* present a major threat to global public health and contribute significantly to morbidity and mortality worldwide. Antibiotic treatment is essential for managing patients infected with these parasites. There is an urgent unmet need to develop effective therapies, given the lack of vaccines, drug resistance, limited efficacy, and toxicity associated with currently available therapies (Field et al., 2017; De Rycker et al., 2018; Shirley et al., 2019). Repurposing existing drugs offers advantages over *de novo* drug development including cost and time reduction. Disulfiram is already FDA-approved, with a long history of use in medicine and safety information already at hand, and could progress more rapidly through investigation under a repurposed indication. Also, disulfiram is already being repurposed for treating cancer patients. Furthermore, disulfiram is widely available, has a good safety profile, and is an economical and low-cost drug, which could make it a viable economic option for patients in low-income settings (Cvek, 2012). Affordability in low-income countries is an important factor to consider, as parasitic infections such as amebiasis are most prevalent in poverty-stricken settings. Future acceleration to phase II randomized control trials will allow further investigation of the microbiologic and clinical efficacy of disulfiram as an anti-parasitic.

## Data Availability Statement

The original contributions presented in the study are included in the article/supplementary material. Further inquiries can be directed to the corresponding author.

## Author Contributions

D-AS, IS, CW, and SM wrote, edited and/or reviewed the manuscript. All authors contributed to the article and approved the submitted version.

## Funding

This work was supported by NIH R01AI026649, K08AI119181, and the Robert Wood Johnson Foundation–Harold Amos Medical Faculty Development Program Award.

## Conflict of Interest

The authors declare that the research was conducted in the absence of any commercial or financial relationships that could be construed as a potential conflict of interest.
